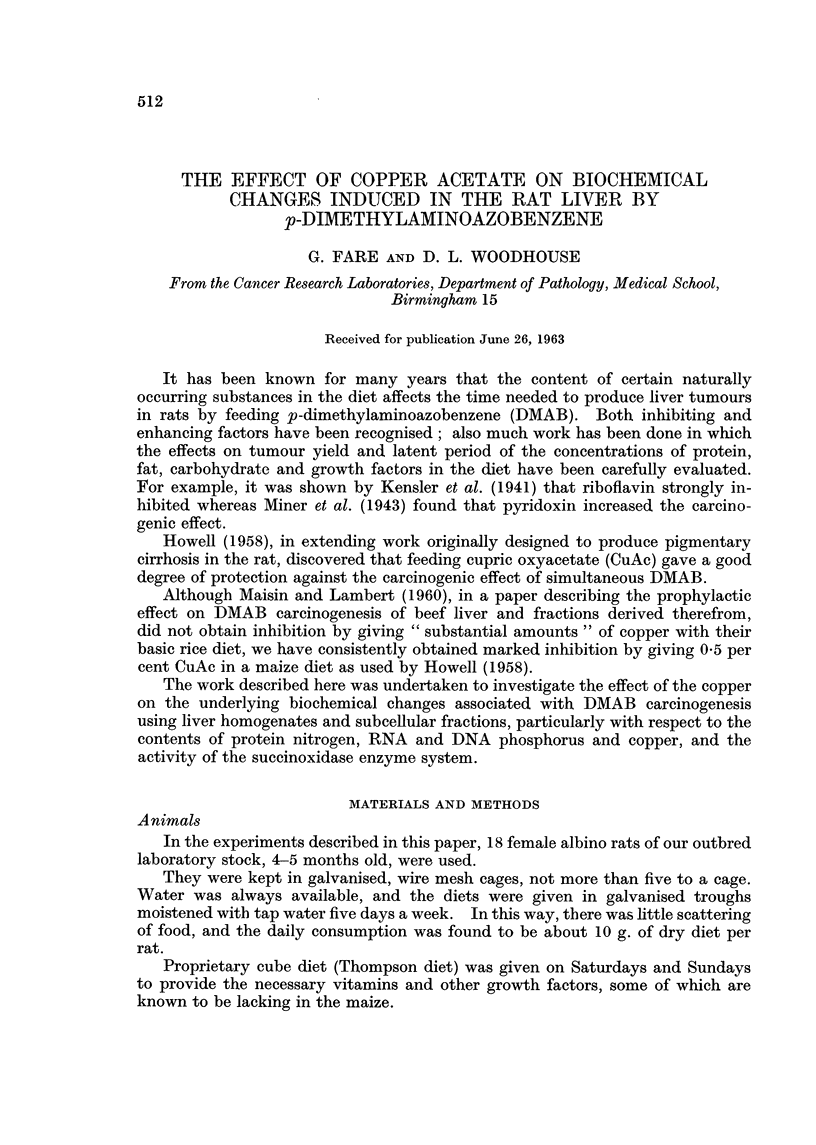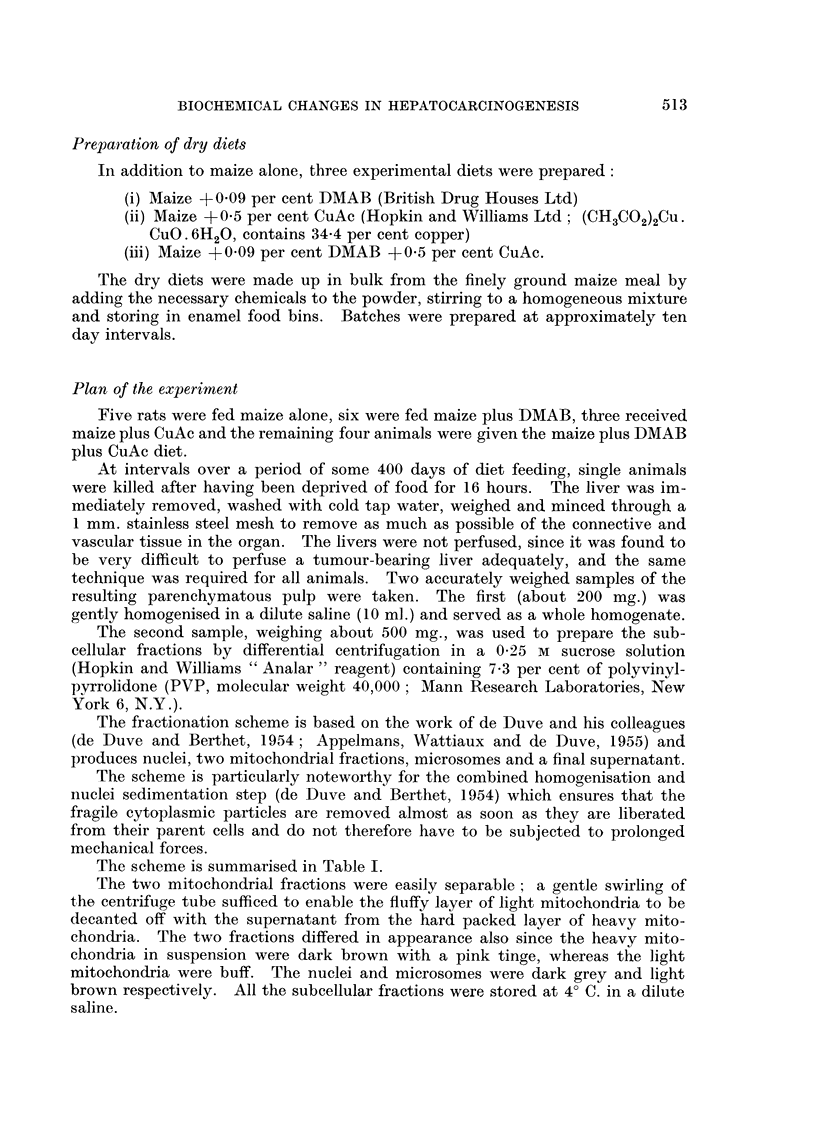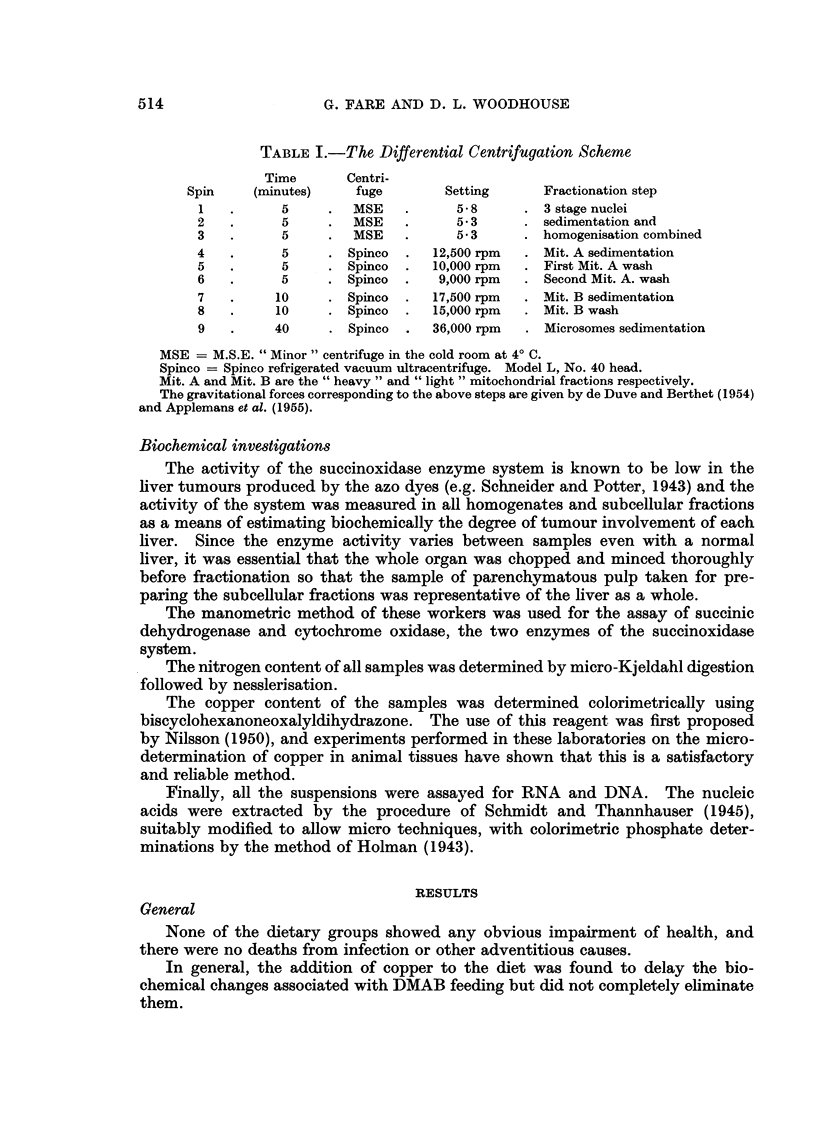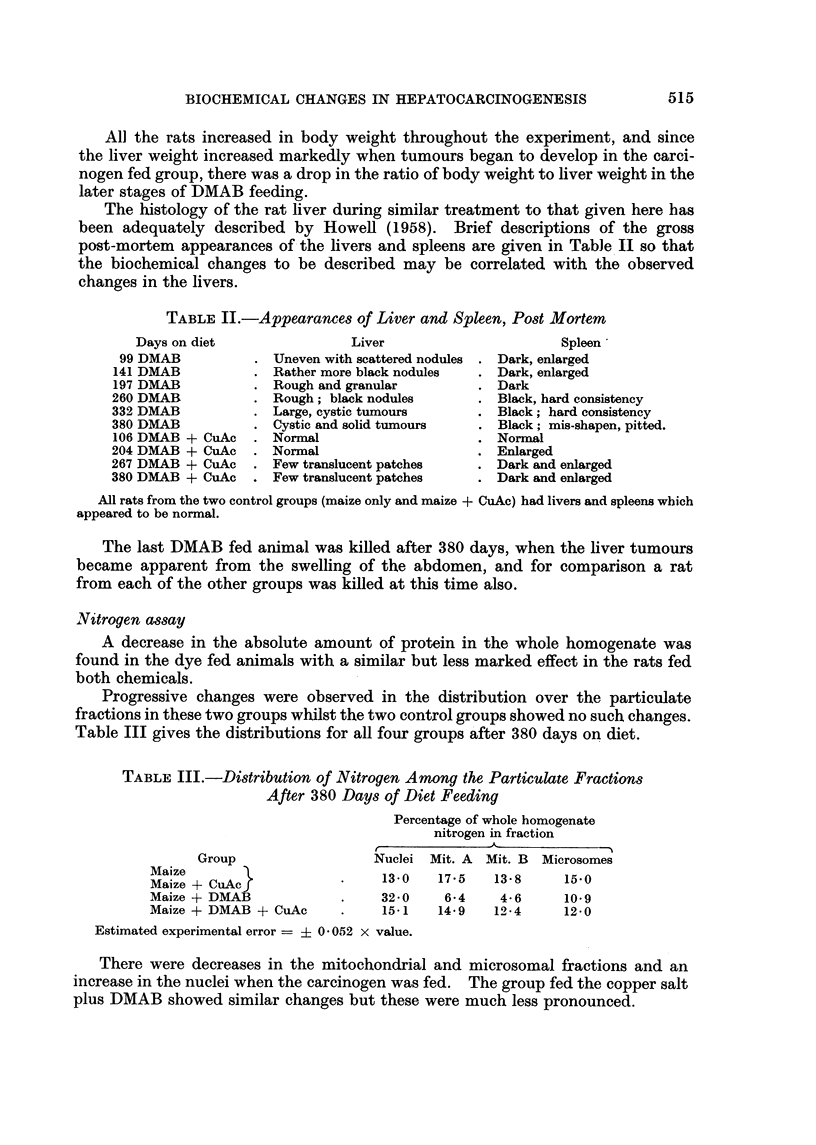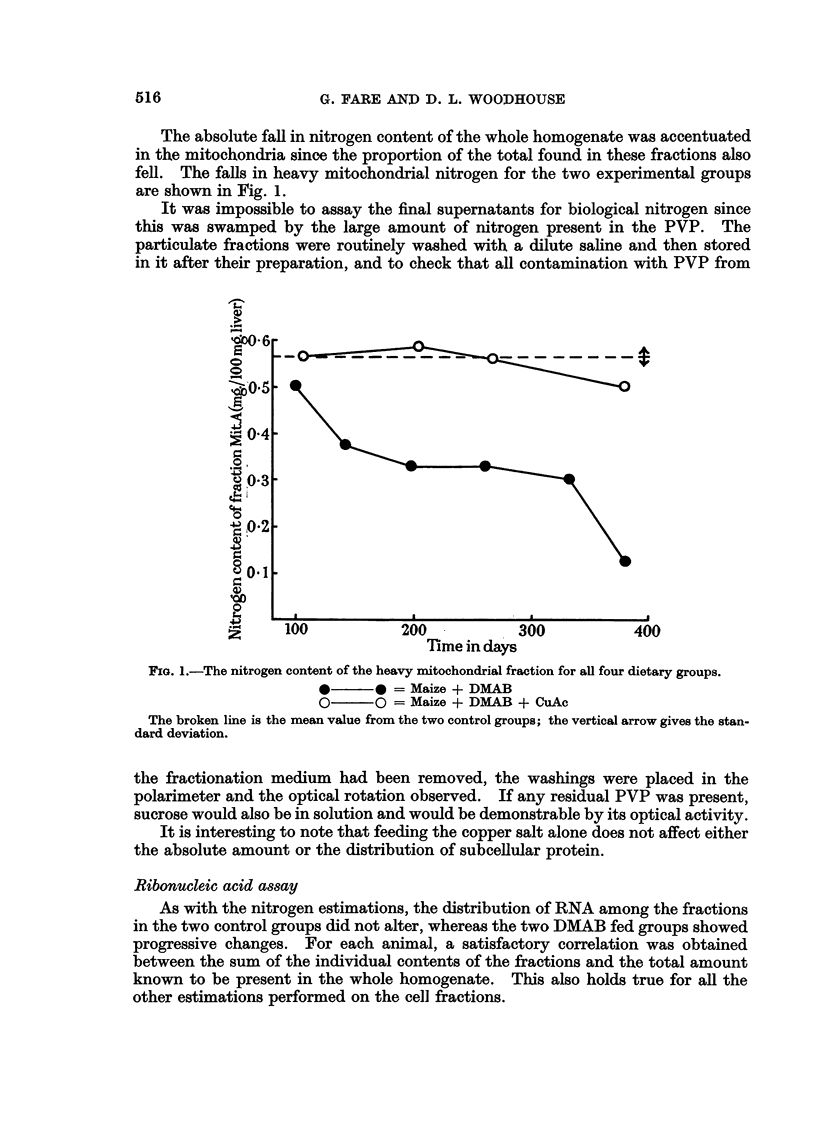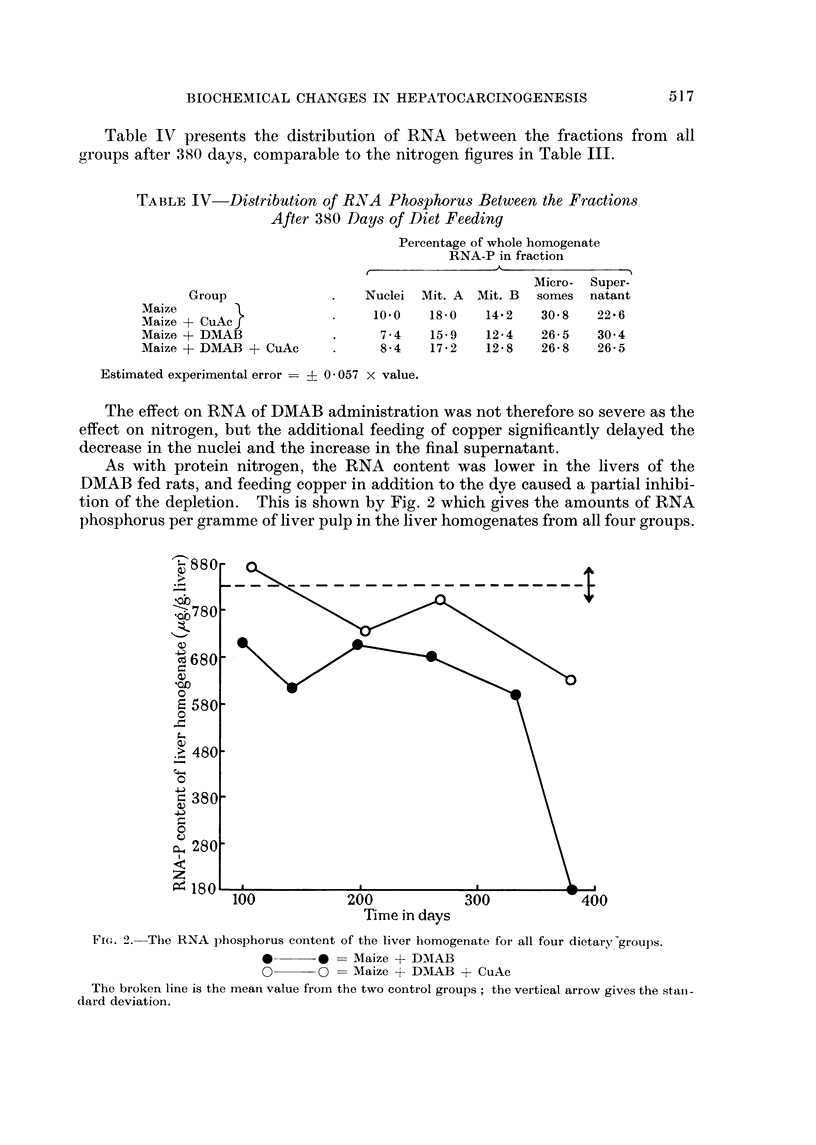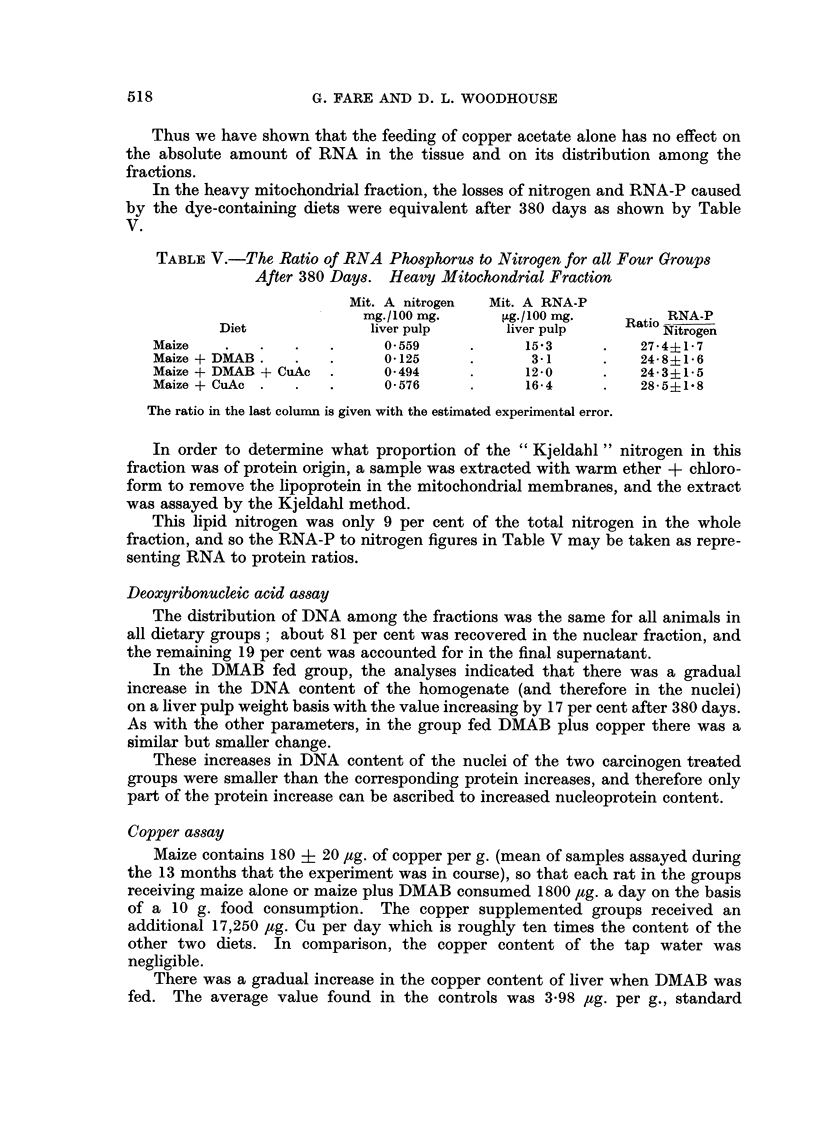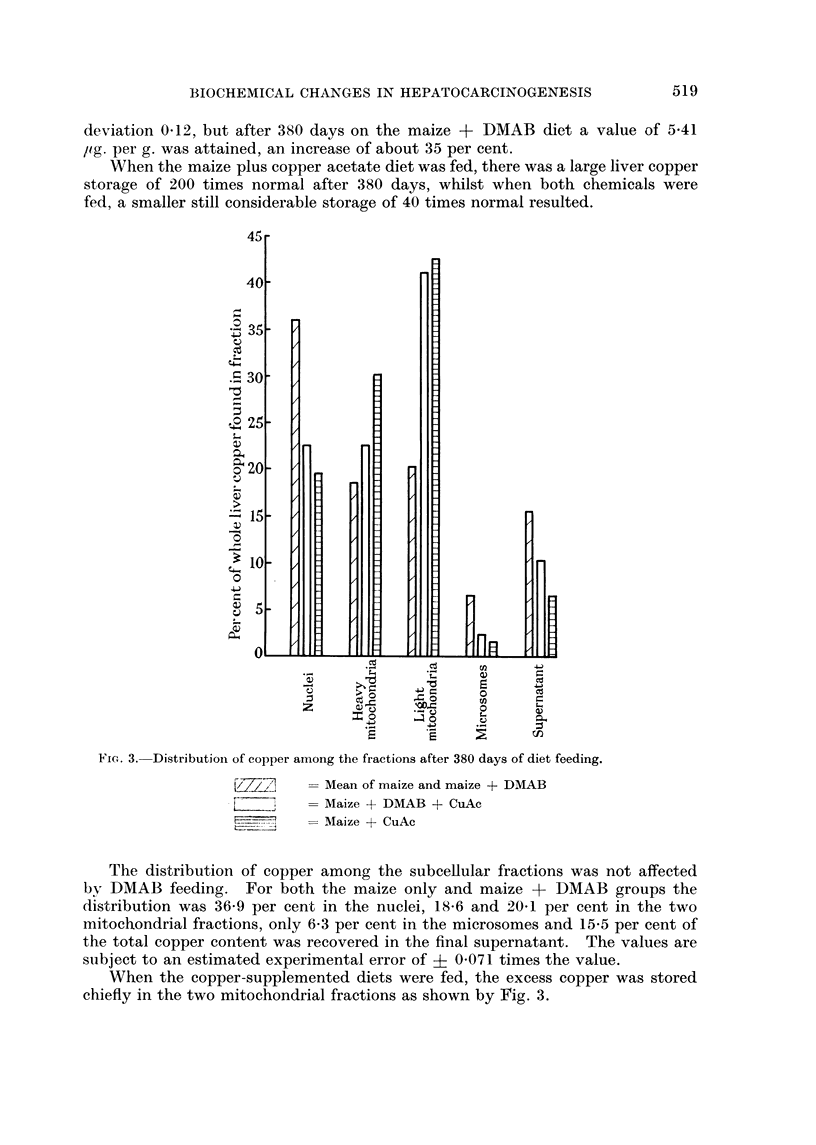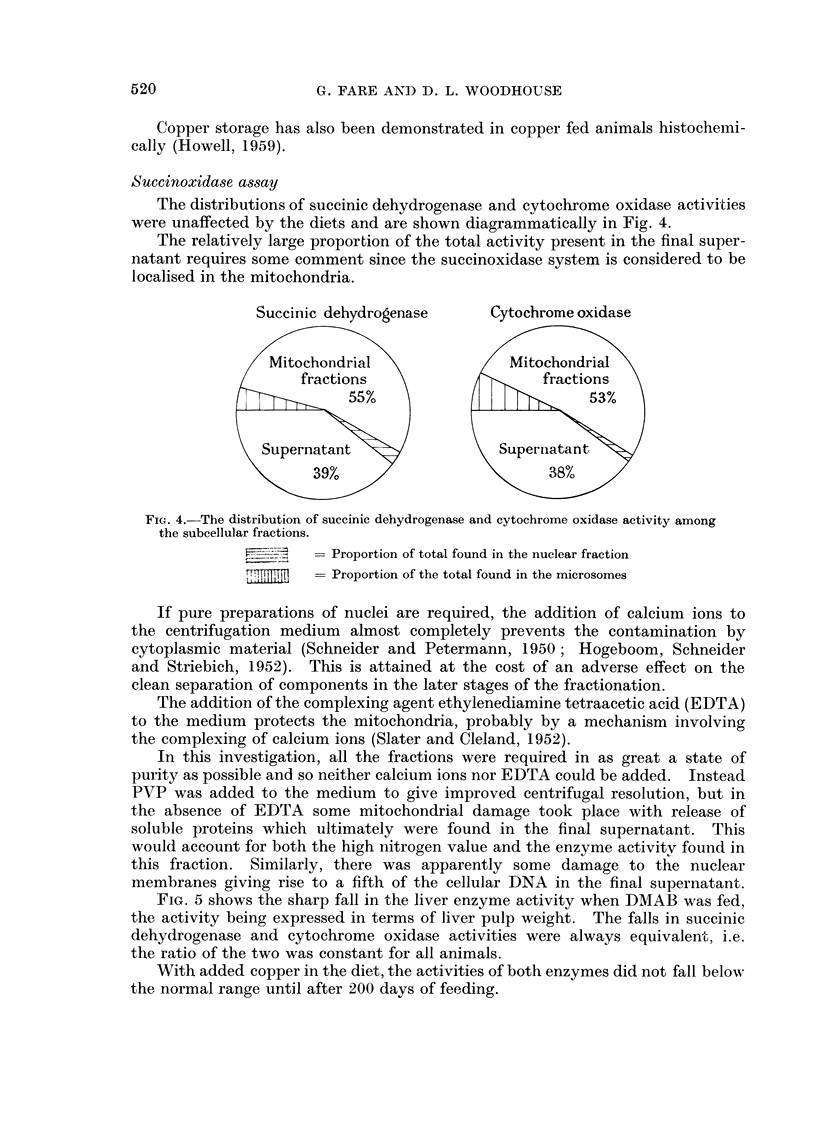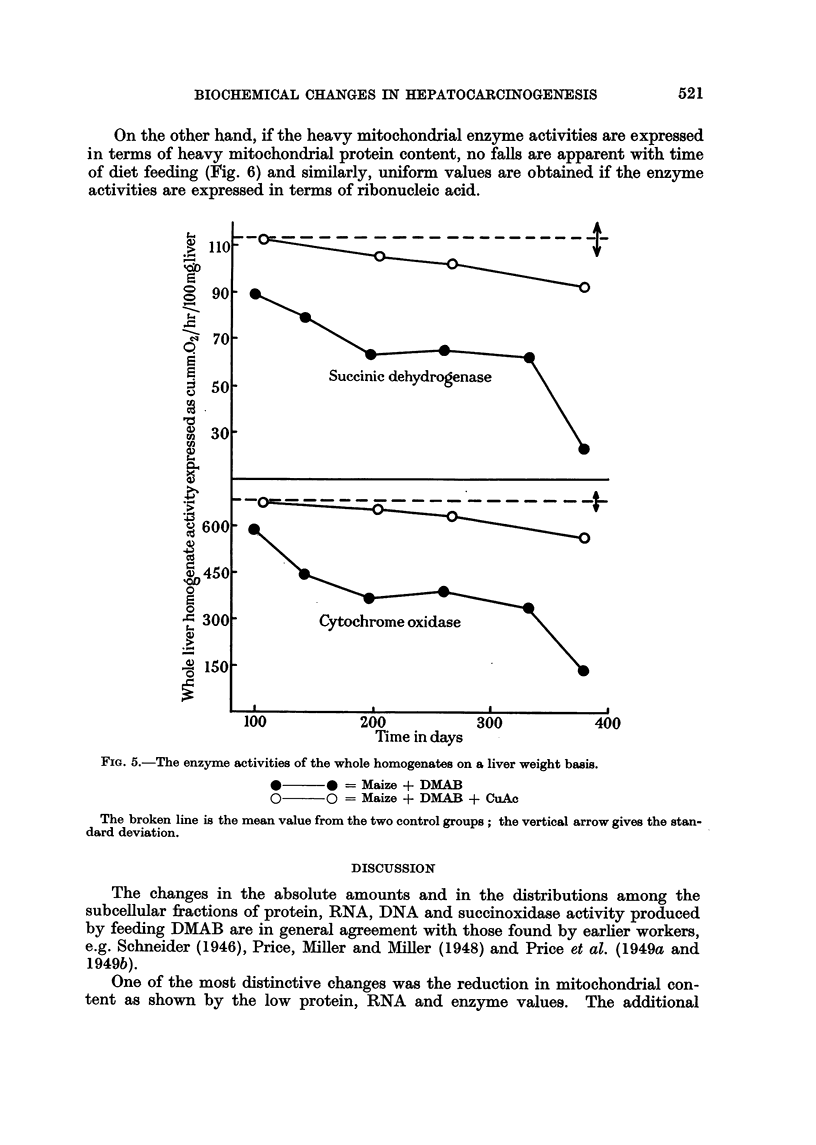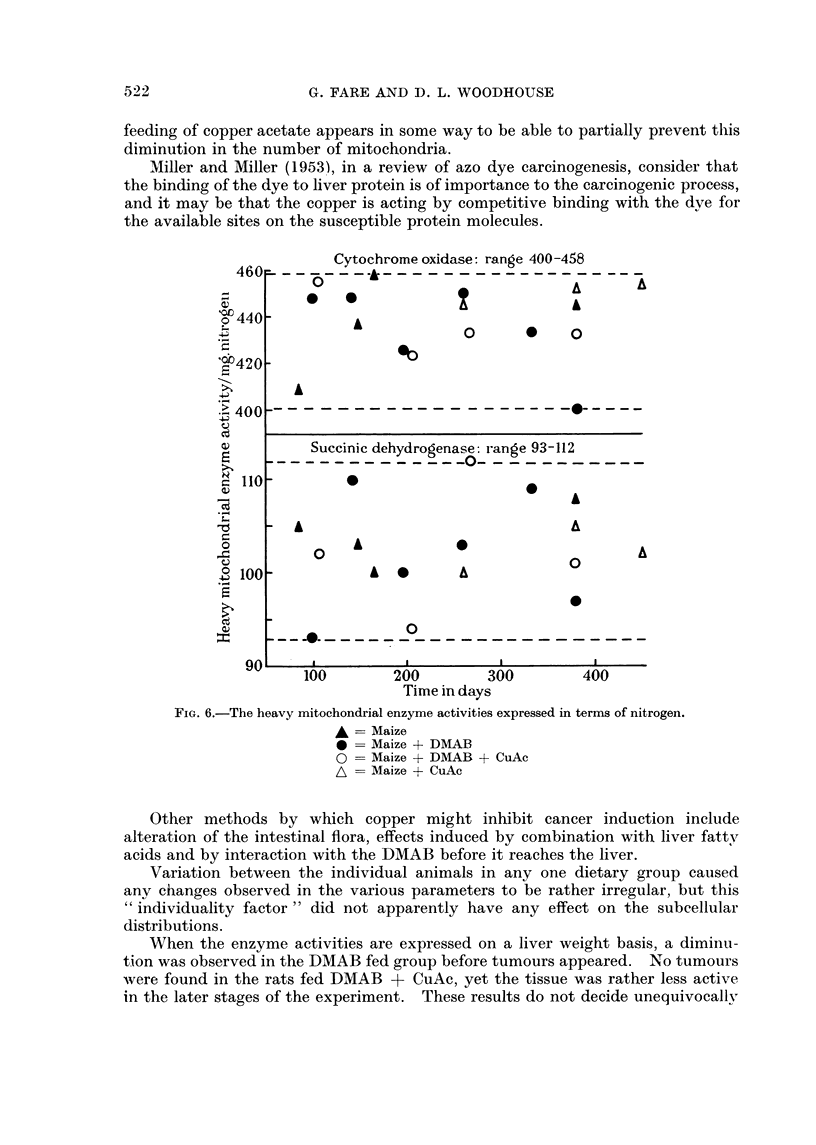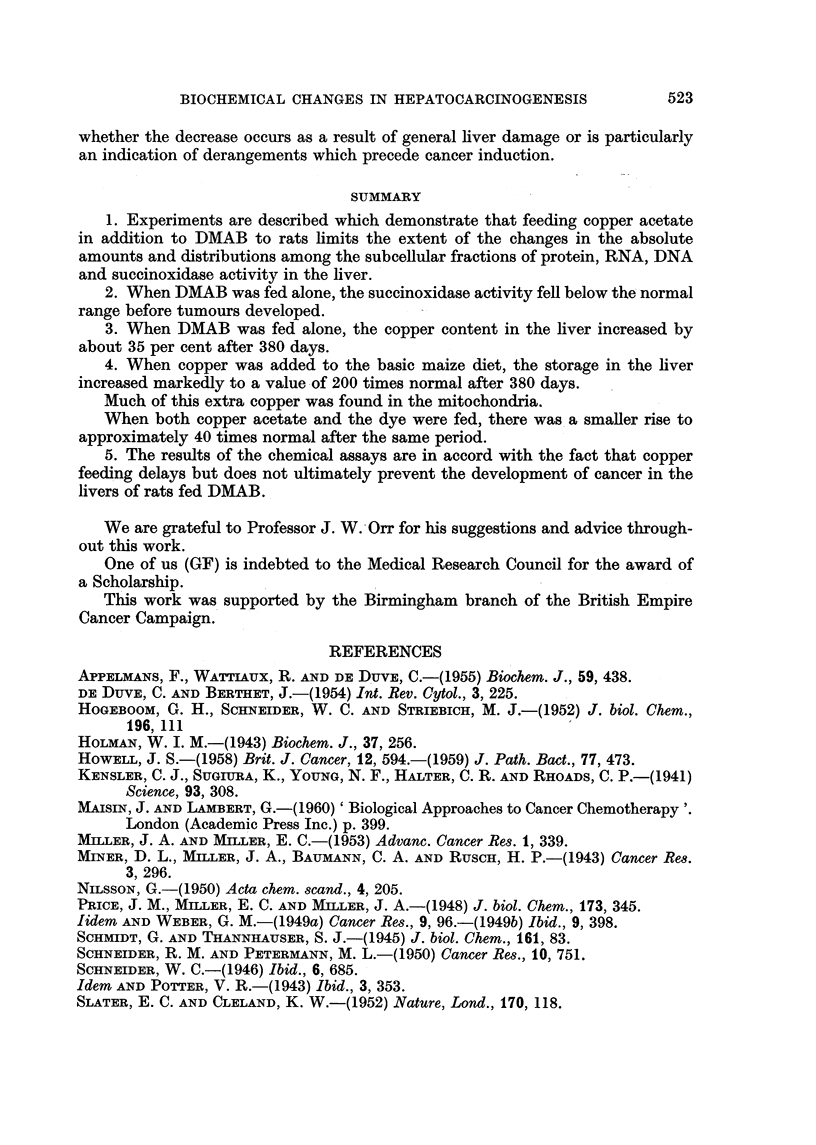# The Effect of Copper Acetate on Biochemical Changes Induced in the Rat Liver by p-Dimethylaminoazobenzene

**DOI:** 10.1038/bjc.1963.69

**Published:** 1963-09

**Authors:** G. Fare, D. L. Woodhouse


### THE EFFECT OF COPPER ACETATE ON BIOCHEMICAL CHANGES INDUCED IN THE RAT LIVER BY *p*-DIMETHYLAMINOAZOBENZENE

G. FARE AND D. L. WOODHOUSE

*From the Cancer Research Laboratories, Department of Pathology, Medical School, Birmingham* 15

Received for publication June 26, 1963

It has been known for many years that the content of certain naturally occurring substances in the diet affects the time needed to produce liver tumours in rats by feeding *p*-dimethylaminoazobenzene (DMAB). Both inhibiting and enhancing factors have been recognised ; also much work has been done in which the effects on tumour yield and latent period of the concentrations of protein, fat, carbohydrate and growth factors in the diet have been carefully evaluated. For example, it was shown by Kensler *et al.* (1941) that riboflavin strongly inhibited whereas Miner *et al.* (1943) found that pyridoxin increased the carcinogenic effect.

Howell (1958), in extending work originally designed to produce pigmentary cirrhosis in the rat, discovered that feeding cupric oxyacetate (CuAc) gave a good degree of protection against the carcinogenic effect of simultaneous DMAB.

Although Maisin and Lambert (1960), in a paper describing the prophylactic effect on DMAB carcinogenesis of beef liver and fractions derived therefrom, did not obtain inhibition by giving " substantial amounts " of copper with their basic rice diet, we have consistently obtained marked inhibition by giving 0·5 per cent CuAc in a maize diet as used by Howell (1958).

The work described here was undertaken to investigate the effect of the copper on the underlying biochemical changes associated with DMAB carcinogenesis using liver homogenates and subcellular fractions, particularly with respect to the contents of protein nitrogen, RNA and DNA phosphorus and copper, and the activity of the succinoxidase enzyme system.

### MATERIALS AND METHODS

### *Animals*

In the experiments described in this paper, 18 female albino rats of our outbred laboratory stock, 4–5 months old, were used.

They were kept in galvanised, wire mesh cages, not more than five to a cage. Water was always available, and the diets were given in galvanised troughs moistened with tap water five days a week. In this way, there was little scattering of food, and the daily consumption was found to be about 10 g. of dry diet per rat.

Proprietary cube diet (Thompson diet) was given on Saturdays and Sundays to provide the necessary vitamins and other growth factors, some of which are known to be lacking in the maize.

*Preparation of dry diets*

In addition to maize alone, three experimental diets were prepared :

(i) Maize +0·09 per cent DMAB (British Drug Houses Ltd)
(ii) Maize +0·5 per cent CuAc (Hopkin and Williams Ltd ; $(CH_3CO_2)_2Cu.CuO.6H_2O$, contains 34·4 per cent copper)
(iii) Maize +0·09 per cent DMAB +0·5 per cent CuAc.

The dry diets were made up in bulk from the finely ground maize meal by adding the necessary chemicals to the powder, stirring to a homogeneous mixture and storing in enamel food bins. Batches were prepared at approximately ten day intervals.

*Plan of the experiment*

Five rats were fed maize alone, six were fed maize plus DMAB, three received maize plus CuAc and the remaining four animals were given the maize plus DMAB plus CuAc diet.

At intervals over a period of some 400 days of diet feeding, single animals were killed after having been deprived of food for 16 hours. The liver was immediately removed, washed with cold tap water, weighed and minced through a 1 mm. stainless steel mesh to remove as much as possible of the connective and vascular tissue in the organ. The livers were not perfused, since it was found to be very difficult to perfuse a tumour-bearing liver adequately, and the same technique was required for all animals. Two accurately weighed samples of the resulting parenchymatous pulp were taken. The first (about 200 mg.) was gently homogenised in a dilute saline (10 ml.) and served as a whole homogenate.

The second sample, weighing about 500 mg., was used to prepare the subcellular fractions by differential centrifugation in a 0·25 M sucrose solution (Hopkin and Williams "Analar" reagent) containing 7·3 per cent of polyvinylpyrrolidone (PVP, molecular weight 40,000 ; Mann Research Laboratories, New York 6, N.Y.).

The fractionation scheme is based on the work of de Duve and his colleagues (de Duve and Berthet, 1954 ; Appelmans, Wattiaux and de Duve, 1955) and produces nuclei, two mitochondrial fractions, microsomes and a final supernatant.

The scheme is particularly noteworthy for the combined homogenisation and nuclei sedimentation step (de Duve and Berthet, 1954) which ensures that the fragile cytoplasmic particles are removed almost as soon as they are liberated from their parent cells and do not therefore have to be subjected to prolonged mechanical forces.

The scheme is summarised in Table I.

The two mitochondrial fractions were easily separable ; a gentle swirling of the centrifuge tube sufficed to enable the fluffy layer of light mitochondria to be decanted off with the supernatant from the hard packed layer of heavy mitochondria. The two fractions differed in appearance also since the heavy mitochondria in suspension were dark brown with a pink tinge, whereas the light mitochondria were buff. The nuclei and microsomes were dark grey and light brown respectively. All the subcellular fractions were stored at 4° C. in a dilute saline.

TABLE I.—*The Differential Centrifugation Scheme*

| Spin | Time (minutes) | Centri-fuge | Setting | Fractionation step |
|---|---|---|---|---|
| 1 | 5 | MSE | 5·8 | 3 stage nuclei |
| 2 | 5 | MSE | 5·3 | sedimentation and |
| 3 | 5 | MSE | 5·3 | homogenisation combined |
| 4 | 5 | Spinco | 12,500 rpm | Mit. A sedimentation |
| 5 | 5 | Spinco | 10,000 rpm | First Mit. A wash |
| 6 | 5 | Spinco | 9,000 rpm | Second Mit. A. wash |
| 7 | 10 | Spinco | 17,500 rpm | Mit. B sedimentation |
| 8 | 10 | Spinco | 15,000 rpm | Mit. B wash |
| 9 | 40 | Spinco | 36,000 rpm | Microsomes sedimentation |

MSE = M.S.E. " Minor " centrifuge in the cold room at 4° C.
Spinco = Spinco refrigerated vacuum ultracentrifuge. Model L, No. 40 head.
Mit. A and Mit. B are the " heavy " and " light " mitochondrial fractions respectively.
The gravitational forces corresponding to the above steps are given by de Duve and Berthet (1954) and Applemans *et al.* (1955).

*Biochemical investigations*

The activity of the succinoxidase enzyme system is known to be low in the liver tumours produced by the azo dyes (e.g. Schneider and Potter, 1943) and the activity of the system was measured in all homogenates and subcellular fractions as a means of estimating biochemically the degree of tumour involvement of each liver. Since the enzyme activity varies between samples even with a normal liver, it was essential that the whole organ was chopped and minced thoroughly before fractionation so that the sample of parenchymatous pulp taken for preparing the subcellular fractions was representative of the liver as a whole.

The manometric method of these workers was used for the assay of succinic dehydrogenase and cytochrome oxidase, the two enzymes of the succinoxidase system.

The nitrogen content of all samples was determined by micro-Kjeldahl digestion followed by nesslerisation.

The copper content of the samples was determined colorimetrically using biscyclohexanoneoxalyldihydrazone. The use of this reagent was first proposed by Nilsson (1950), and experiments performed in these laboratories on the microdetermination of copper in animal tissues have shown that this is a satisfactory and reliable method.

Finally, all the suspensions were assayed for RNA and DNA. The nucleic acids were extracted by the procedure of Schmidt and Thannhauser (1945), suitably modified to allow micro techniques, with colorimetric phosphate determinations by the method of Holman (1943).

### RESULTS

*General*

None of the dietary groups showed any obvious impairment of health, and there were no deaths from infection or other adventitious causes.

In general, the addition of copper to the diet was found to delay the biochemical changes associated with DMAB feeding but did not completely eliminate them.

All the rats increased in body weight throughout the experiment, and since the liver weight increased markedly when tumours began to develop in the carcinogen fed group, there was a drop in the ratio of body weight to liver weight in the later stages of DMAB feeding.

The histology of the rat liver during similar treatment to that given here has been adequately described by Howell (1958). Brief descriptions of the gross post-mortem appearances of the livers and spleens are given in Table II so that the biochemical changes to be described may be correlated with the observed changes in the livers.

TABLE II.—*Appearances of Liver and Spleen, Post Mortem*

| Days on diet | Liver | Spleen |
|---|---|---|
| 99 DMAB | Uneven with scattered nodules | Dark, enlarged |
| 141 DMAB | Rather more black nodules | Dark, enlarged |
| 197 DMAB | Rough and granular | Dark |
| 260 DMAB | Rough ; black nodules | Black, hard consistency |
| 332 DMAB | Large, cystic tumours | Black ; hard consistency |
| 380 DMAB | Cystic and solid tumours | Black ; mis-shapen, pitted. |
| 106 DMAB + CuAc | Normal | Normal |
| 204 DMAB + CuAc | Normal | Enlarged |
| 267 DMAB + CuAc | Few translucent patches | Dark and enlarged |
| 380 DMAB + CuAc | Few translucent patches | Dark and enlarged |

All rats from the two control groups (maize only and maize + CuAc) had livers and spleens which appeared to be normal.

The last DMAB fed animal was killed after 380 days, when the liver tumours became apparent from the swelling of the abdomen, and for comparison a rat from each of the other groups was killed at this time also.

*Nitrogen assay*

A decrease in the absolute amount of protein in the whole homogenate was found in the dye fed animals with a similar but less marked effect in the rats fed both chemicals.

Progressive changes were observed in the distribution over the particulate fractions in these two groups whilst the two control groups showed no such changes. Table III gives the distributions for all four groups after 380 days on diet.

TABLE III.—*Distribution of Nitrogen Among the Particulate Fractions After* 380 *Days of Diet Feeding*

| Group | Percentage of whole homogenate nitrogen in fraction | | | |
|---|---|---|---|---|
| | Nuclei | Mit. A | Mit. B | Microsomes |
| Maize; Maize + CuAc | 13·0 | 17·5 | 13·8 | 15·0 |
| Maize + DMAB | 32·0 | 6·4 | 4·6 | 10·9 |
| Maize + DMAB + CuAc | 15·1 | 14·9 | 12·4 | 12·0 |

Estimated experimental error = $\pm$ 0·052 $\times$ value.

There were decreases in the mitochondrial and microsomal fractions and an increase in the nuclei when the carcinogen was fed. The group fed the copper salt plus DMAB showed similar changes but these were much less pronounced.

The absolute fall in nitrogen content of the whole homogenate was accentuated in the mitochondria since the proportion of the total found in these fractions also fell. The falls in heavy mitochondrial nitrogen for the two experimental groups are shown in Fig. 1.

It was impossible to assay the final supernatants for biological nitrogen since this was swamped by the large amount of nitrogen present in the PVP. The particulate fractions were routinely washed with a dilute saline and then stored in it after their preparation, and to check that all contamination with PVP from

**Fig. 1.**—The nitrogen content of the heavy mitochondrial fraction for all four dietary groups.

●———● = Maize + DMAB
○———○ = Maize + DMAB + CuAc

The broken line is the mean value from the two control groups; the vertical arrow gives the standard deviation.

the fractionation medium had been removed, the washings were placed in the polarimeter and the optical rotation observed. If any residual PVP was present, sucrose would also be in solution and would be demonstrable by its optical activity.

It is interesting to note that feeding the copper salt alone does not affect either the absolute amount or the distribution of subcellular protein.

*Ribonucleic acid assay*

As with the nitrogen estimations, the distribution of RNA among the fractions in the two control groups did not alter, whereas the two DMAB fed groups showed progressive changes. For each animal, a satisfactory correlation was obtained between the sum of the individual contents of the fractions and the total amount known to be present in the whole homogenate. This also holds true for all the other estimations performed on the cell fractions.

Table IV presents the distribution of RNA between the fractions from all groups after 380 days, comparable to the nitrogen figures in Table III.

TABLE IV—*Distribution of RNA Phosphorus Between the Fractions After* 380 *Days of Diet Feeding*

| Group | Percentage of whole homogenate RNA-P in fraction: Nuclei | Mit. A | Mit. B | Micro-somes | Super-natant |
|---|---|---|---|---|---|
| Maize; Maize + CuAc | 10·0 | 18·0 | 14·2 | 30·8 | 22·6 |
| Maize + DMAB | 7·4 | 15·9 | 12·4 | 26·5 | 30·4 |
| Maize + DMAB + CuAc | 8·4 | 17·2 | 12·8 | 26·8 | 26·5 |

Estimated experimental error = ± 0·057 × value.

The effect on RNA of DMAB administration was not therefore so severe as the effect on nitrogen, but the additional feeding of copper significantly delayed the decrease in the nuclei and the increase in the final supernatant.

As with protein nitrogen, the RNA content was lower in the livers of the DMAB fed rats, and feeding copper in addition to the dye caused a partial inhibition of the depletion. This is shown by Fig. 2 which gives the amounts of RNA phosphorus per gramme of liver pulp in the liver homogenates from all four groups.

FIG. 2.—The RNA phosphorus content of the liver homogenate for all four dietary groups.

●———● = Maize + DMAB
○———○ = Maize + DMAB + CuAc

The broken line is the mean value from the two control groups ; the vertical arrow gives the standard deviation.

Thus we have shown that the feeding of copper acetate alone has no effect on the absolute amount of RNA in the tissue and on its distribution among the fractions.

In the heavy mitochondrial fraction, the losses of nitrogen and RNA-P caused by the dye-containing diets were equivalent after 380 days as shown by Table V.

TABLE V.—*The Ratio of RNA Phosphorus to Nitrogen for all Four Groups After* 380 *Days. Heavy Mitochondrial Fraction*

| Diet | Mit. A nitrogen mg./100 mg. liver pulp | Mit. A RNA-P µg./100 mg. liver pulp | Ratio $\frac{\text{RNA-P}}{\text{Nitrogen}}$ |
|---|---|---|---|
| Maize | 0·559 | 15·3 | 27·4±1·7 |
| Maize + DMAB | 0·125 | 3·1 | 24·8±1·6 |
| Maize + DMAB + CuAc | 0·494 | 12·0 | 24·3±1·5 |
| Maize + CuAc | 0·576 | 16·4 | 28·5±1·8 |

The ratio in the last column is given with the estimated experimental error.

In order to determine what proportion of the "Kjeldahl" nitrogen in this fraction was of protein origin, a sample was extracted with warm ether + chloroform to remove the lipoprotein in the mitochondrial membranes, and the extract was assayed by the Kjeldahl method.

This lipid nitrogen was only 9 per cent of the total nitrogen in the whole fraction, and so the RNA-P to nitrogen figures in Table V may be taken as representing RNA to protein ratios.

*Deoxyribonucleic acid assay*

The distribution of DNA among the fractions was the same for all animals in all dietary groups; about 81 per cent was recovered in the nuclear fraction, and the remaining 19 per cent was accounted for in the final supernatant.

In the DMAB fed group, the analyses indicated that there was a gradual increase in the DNA content of the homogenate (and therefore in the nuclei) on a liver pulp weight basis with the value increasing by 17 per cent after 380 days. As with the other parameters, in the group fed DMAB plus copper there was a similar but smaller change.

These increases in DNA content of the nuclei of the two carcinogen treated groups were smaller than the corresponding protein increases, and therefore only part of the protein increase can be ascribed to increased nucleoprotein content.

*Copper assay*

Maize contains 180 ± 20 µg. of copper per g. (mean of samples assayed during the 13 months that the experiment was in course), so that each rat in the groups receiving maize alone or maize plus DMAB consumed 1800 µg. a day on the basis of a 10 g. food consumption. The copper supplemented groups received an additional 17,250 µg. Cu per day which is roughly ten times the content of the other two diets. In comparison, the copper content of the tap water was negligible.

There was a gradual increase in the copper content of liver when DMAB was fed. The average value found in the controls was 3·98 µg. per g., standard deviation 0·12, but after 380 days on the maize + DMAB diet a value of 5·41 μg. per g. was attained, an increase of about 35 per cent.

When the maize plus copper acetate diet was fed, there was a large liver copper storage of 200 times normal after 380 days, whilst when both chemicals were fed, a smaller still considerable storage of 40 times normal resulted.

FIG. 3.—Distribution of copper among the fractions after 380 days of diet feeding.

(hatched) = Mean of maize and maize + DMAB

(open) = Maize + DMAB + CuAc

(lined) = Maize + CuAc

The distribution of copper among the subcellular fractions was not affected by DMAB feeding. For both the maize only and maize + DMAB groups the distribution was 36·9 per cent in the nuclei, 18·6 and 20·1 per cent in the two mitochondrial fractions, only 6·3 per cent in the microsomes and 15·5 per cent of the total copper content was recovered in the final supernatant. The values are subject to an estimated experimental error of ± 0·071 times the value.

When the copper-supplemented diets were fed, the excess copper was stored chiefly in the two mitochondrial fractions as shown by Fig. 3.

Copper storage has also been demonstrated in copper fed animals histochemically (Howell, 1959).

*Succinoxidase assay*

The distributions of succinic dehydrogenase and cytochrome oxidase activities were unaffected by the diets and are shown diagrammatically in Fig. 4.

The relatively large proportion of the total activity present in the final supernatant requires some comment since the succinoxidase system is considered to be localised in the mitochondria.

Fig. 4.—The distribution of succinic dehydrogenase and cytochrome oxidase activity among the subcellular fractions.

 = Proportion of total found in the nuclear fraction

 = Proportion of the total found in the microsomes

If pure preparations of nuclei are required, the addition of calcium ions to the centrifugation medium almost completely prevents the contamination by cytoplasmic material (Schneider and Petermann, 1950 ; Hogeboom, Schneider and Striebich, 1952). This is attained at the cost of an adverse effect on the clean separation of components in the later stages of the fractionation.

The addition of the complexing agent ethylenediamine tetraacetic acid (EDTA) to the medium protects the mitochondria, probably by a mechanism involving the complexing of calcium ions (Slater and Cleland, 1952).

In this investigation, all the fractions were required in as great a state of purity as possible and so neither calcium ions nor EDTA could be added. Instead PVP was added to the medium to give improved centrifugal resolution, but in the absence of EDTA some mitochondrial damage took place with release of soluble proteins which ultimately were found in the final supernatant. This would account for both the high nitrogen value and the enzyme activity found in this fraction. Similarly, there was apparently some damage to the nuclear membranes giving rise to a fifth of the cellular DNA in the final supernatant.

Fig. 5 shows the sharp fall in the liver enzyme activity when DMAB was fed, the activity being expressed in terms of liver pulp weight. The falls in succinic dehydrogenase and cytochrome oxidase activities were always equivalent, i.e. the ratio of the two was constant for all animals.

With added copper in the diet, the activities of both enzymes did not fall below the normal range until after 200 days of feeding.

On the other hand, if the heavy mitochondrial enzyme activities are expressed in terms of heavy mitochondrial protein content, no falls are apparent with time of diet feeding (Fig. 6) and similarly, uniform values are obtained if the enzyme activities are expressed in terms of ribonucleic acid.

FIG. 5.—The enzyme activities of the whole homogenates on a liver weight basis.

●———● = Maize + DMAB
○———○ = Maize + DMAB + CuAc

The broken line is the mean value from the two control groups; the vertical arrow gives the standard deviation.

### DISCUSSION

The changes in the absolute amounts and in the distributions among the subcellular fractions of protein, RNA, DNA and succinoxidase activity produced by feeding DMAB are in general agreement with those found by earlier workers, e.g. Schneider (1946), Price, Miller and Miller (1948) and Price *et al.* (1949*a* and 1949*b*).

One of the most distinctive changes was the reduction in mitochondrial content as shown by the low protein, RNA and enzyme values. The additional feeding of copper acetate appears in some way to be able to partially prevent this diminution in the number of mitochondria.

Miller and Miller (1953), in a review of azo dye carcinogenesis, consider that the binding of the dye to liver protein is of importance to the carcinogenic process, and it may be that the copper is acting by competitive binding with the dye for the available sites on the susceptible protein molecules.

Fig. 6.—The heavy mitochondrial enzyme activities expressed in terms of nitrogen.

▲ = Maize
● = Maize + DMAB
○ = Maize + DMAB + CuAc
△ = Maize + CuAc

Other methods by which copper might inhibit cancer induction include alteration of the intestinal flora, effects induced by combination with liver fatty acids and by interaction with the DMAB before it reaches the liver.

Variation between the individual animals in any one dietary group caused any changes observed in the various parameters to be rather irregular, but this " individuality factor " did not apparently have any effect on the subcellular distributions.

When the enzyme activities are expressed on a liver weight basis, a diminution was observed in the DMAB fed group before tumours appeared. No tumours were found in the rats fed DMAB + CuAc, yet the tissue was rather less active in the later stages of the experiment. These results do not decide unequivocally whether the decrease occurs as a result of general liver damage or is particularly an indication of derangements which precede cancer induction.

SUMMARY

1. Experiments are described which demonstrate that feeding copper acetate in addition to DMAB to rats limits the extent of the changes in the absolute amounts and distributions among the subcellular fractions of protein, RNA, DNA and succinoxidase activity in the liver.

2. When DMAB was fed alone, the succinoxidase activity fell below the normal range before tumours developed.

3. When DMAB was fed alone, the copper content in the liver increased by about 35 per cent after 380 days.

4. When copper was added to the basic maize diet, the storage in the liver increased markedly to a value of 200 times normal after 380 days.

Much of this extra copper was found in the mitochondria.

When both copper acetate and the dye were fed, there was a smaller rise to approximately 40 times normal after the same period.

5. The results of the chemical assays are in accord with the fact that copper feeding delays but does not ultimately prevent the development of cancer in the livers of rats fed DMAB.

We are grateful to Professor J. W. Orr for his suggestions and advice throughout this work.

One of us (GF) is indebted to the Medical Research Council for the award of a Scholarship.

This work was supported by the Birmingham branch of the British Empire Cancer Campaign.

REFERENCES

Appelmans, F., Wattiaux, R. and de Duve, C.—(1955) *Biochem. J.*, **59**, 438.

de Duve, C. and Berthet, J.—(1954) *Int. Rev. Cytol.*, **3**, 225.

Hogeboom, G. H., Schneider, W. C. and Striebich, M. J.—(1952) *J. biol. Chem.*, **196**, 111

Holman, W. I. M.—(1943) *Biochem. J.*, **37**, 256.

Howell, J. S.—(1958) *Brit. J. Cancer*, **12**, 594.—(1959) *J. Path. Bact.*, **77**, 473.

Kensler, C. J., Sugiura, K., Young, N. F., Halter, C. R. and Rhoads, C. P.—(1941) *Science*, **93**, 308.

Maisin, J. and Lambert, G.—(1960) ' Biological Approaches to Cancer Chemotherapy '. London (Academic Press Inc.) p. 399.

Miller, J. A. and Miller, E. C.—(1953) *Advanc. Cancer Res.* **1**, 339.

Miner, D. L., Miller, J. A., Baumann, C. A. and Rusch, H. P.—(1943) *Cancer Res.* **3**, 296.

Nilsson, G.—(1950) *Acta chem. scand.*, **4**, 205.

Price, J. M., Miller, E. C. and Miller, J. A.—(1948) *J. biol. Chem.*, **173**, 345.

*Iidem* and Weber, G. M.—(1949*a*) *Cancer Res.*, **9**, 96.—(1949*b*) *Ibid.*, **9**, 398.

Schmidt, G. and Thannhauser, S. J.—(1945) *J. biol. Chem.*, **161**, 83.

Schneider, R. M. and Petermann, M. L.—(1950) *Cancer Res.*, **10**, 751.

Schneider, W. C.—(1946) *Ibid.*, **6**, 685.

*Idem* and Potter, V. R.—(1943) *Ibid.*, **3**, 353.

Slater, E. C. and Cleland, K. W.—(1952) *Nature, Lond.*, **170**, 118.